# Synthesis of Group II-VI Semiconductor Nanocrystals via Phosphine Free Method and Their Application in Solution Processed Photovoltaic Devices

**DOI:** 10.3390/nano11082071

**Published:** 2021-08-15

**Authors:** Mingyue Hou, Zhaohua Zhou, Ao Xu, Kening Xiao, Jiakun Li, Donghuan Qin, Wei Xu, Lintao Hou

**Affiliations:** 1 School of Materials Science and Engineering, South China University of Technology, Guangzhou 510640, China; 201830140083@mail.scut.edu.cn (M.H.); 201830320430@mail.scut.edu.cn (Z.Z.); 202020118759@mail.scut.edu.cn (A.X.); 201830320324@mail.scut.edu.cn (K.X.); 201830640170@mail.scut.edu.cn (J.L.); 2 State Key Laboratory of Luminescent Materials & Devices, Institute of Polymer Optoelectronic Materials & Devices, South China University of Technology, Guangzhou 510640, China; 3 Guangdong Provincial Key Laboratory of Optical Fiber Sensing and Communications, Guangzhou Key Laboratory of Vacuum Coating Technologies and New Energy Materials, Siyuan Laboratory, Department of Physics, Jinan University, Guangzhou 510632, China

**Keywords:** group II-VI semiconductor nanocrystals, phosphine free method, solution process, solar cells

## Abstract

Solution-processed CdTe semiconductor nanocrystals (NCs) have exhibited astonishing potential in fabricating low-cost, low materials consumption and highly efficient photovoltaic devices. However, most of the conventional CdTe NCs reported are synthesized through high temperature microemulsion method with high toxic trioctylphosphine tellurite (TOP-Te) or tributylphosphine tellurite (TBP-Te) as tellurium precursor. These hazardous substances used in the fabrication process of CdTe NCs are drawing them back from further application. Herein, we report a phosphine-free method for synthesizing group II-VI semiconductor NCs with alkyl amine and alkyl acid as ligands. Based on various characterizations like UV-vis absorption (UV), transmission electron microscope (TEM), and X-ray diffraction (XRD), among others, the properties of the as-synthesized CdS, CdSe, and CdTe NCs are determined. High-quality semiconductor NCs with easily controlled size and morphology could be fabricated through this phosphine-free method. To further investigate its potential to industrial application, NCs solar cells with device configuration of ITO/ZnO/CdSe/CdTe/Au and ITO/ZnO/CdS/CdTe/Au are fabricated based on NCs synthesized by this method. By optimizing the device fabrication conditions, the champion device exhibited power conversion efficiency (PCE) of 2.28%. This research paves the way for industrial production of low-cost and environmentally friendly NCs photovoltaic devices.

## 1. Introduction

Group II-VI semiconductor nanocrystals (NCs), such as CdS, CdSe, CdTe, etc., have recently attracted much attention due to their tunable direct bandgap and potential application in optoelectronic devices like photodetectors, photovoltaic devices, and light emitting diodes (LEDs) [1,2,3,4,5,6,7,8]. As the desire for clean and renewable energy increases, photovoltaic devices have become one major research hotspot. In this case, solution processed CdTe NCs solar cells is a promising candidate for next generation commercial photovoltaic device for their low cost, low consumption of materials, simple fabricating process, and being suitable for roll-to-roll printing techniques in industrial mass production [9,10,11]. The controllable synthesis process of high-quality II-VI NCs with uniform morphology, composition, and desired crystal structure is of great significance for fabricating efficient photovoltaic devices [12,13]. To date, group II-VI NCs in most of the reported researches are fabricated by the well-known hot injection method. In such a method, cadmium precursor is first dispersed in a high boiling point solvent such as 1-Octadecene (ODE), Trioctylphosphine oxide (TOPO), and oleic acid (OA), among others. After that, Se or Te precursors (chalcogen elements dissolved into alkylphosphines, such as trioctylphosphine (TOP) or tributylphosphine (TBP)) are quickly injected into the cadmium precursor solution at high temperature (>200 °C) [14,15,16,17]. CdS, CdSe, or CdTe NCs with homogeneous size, crystalline, and morphology can be obtained by varying the reaction temperature, ligands, and precursor concentration. However, the alkylphosphines is highly toxic and expensive, making this method a highly environmentally harmful process. Comparing to the S or Se, preparation of the Te precursors is a great challenge for the insolubility and strong metallicity of the Te element [18,19,20]. As a result, the first step towards a green synthesis method for CdS, CdSe, and CdTe NCs is to prepare phosphine-free S, Se, and Te precursors. Previous researches have reported several ways to obtain a phosphine-free Te precursor. One is based on the water phase green synthesis method, which had been well developed in recent years [21,22,23]. In this method, NaHTe was selected as a Te precursor, which was prepared by adding Te powder and NaHB to deionized water and refluxed under N_2_ flow. Water-soluble CdTe or CdSe NCs can be fabricated based on Se and Te precursors obtained by this process. However, photovoltaic devices fabricated from such water-soluble CdTe/CdSe NCs showed lower devices performance, when compared to similar NC solar cells based on organic phase synthesized CdTe/CdSe NCs [24,25,26,27,28,29,30,31,32,33,34]. In addition, as the reactivity of Te precursor is too high, it is difficult to control the size and morphology of the as-synthesized NCs. Recently, Webber et al. [35] developed a novel binary diamine-dithiol solvent mixture. This mixture is capable of dissolving the VI group element, including S, Se, Te, and other six metals. High-quality telluride semiconductor thin films can be formed by using these precursors. Following this, Yao et al. [19] reported a phosphine-free Se and Te precursor prepared by dissolving SeO_2_ and TeO_2_ in dodecanethiol solvent by sonication under low temperature. It was found that SeO_2_ and TeO_2_ are reduced by dodecanethiol to elemental Se and Te, respectively. After combining disulfides, various metal chalcogenide NCs, including CdSe, CdTe, and other tellurite semiconductors can be synthesized. Later, Wu’s group [36] reported a phosphine-free Te precursor prepared by dissolving TeO_2_ into dodecanethiol and oleylamine mixture. CdTe, PbTe, FeTe_2_, and Cu_2_Te NCs with homogeneous size and morphology were obtained by controlling reaction conditions, such as temperature, and precursor concentration. Although the II-VI group semiconductor NCs was fabricated successfully via a phosphine-free environmentally friendly way, there are still no reports on photovoltaic devices’ application based on these semiconductor NCs. 

In this research, we demonstrate a low-cost, efficient phosphine-free method for synthesis of CdS, CdSe, and CdTe NCs in organic solvents. Dodecanethiol and oleic amine were selected as complex ligands for the Te precursor, while Se or S powder was used directly for the synthesis of CdSe or CdS NCs. The morphology and structure of the NCs were characterized by TEM and XRD, respectively, while the UV-vis absorption was used to investigate the optical properties of NCs. Based on the semiconductor NCs synthesized by phosphine-free process, solar cells with configuration of ITO/ZnO/CdS/CdTe/Au and ITO/ZnO/CdSe/CdTe/Au were fabricated through a layer-by-layer solution process. The effects of active layer thickness and annealing temperature on the device’s performance are investigated and discussed in detail. Champion devices with PCE of 1.08% and 2.28% were fabricated when adopting CdSe and CdS as *n*-type materials, respectively. Using this low-cost and low-toxicity synthesis method, this research has shown the potential of manufacturing environmentally friendly, low-cost, and large-area solution-processed CdTe NCs solar cells.

## 2. Experiment Procedure

### 2.1. Materials

Anhydrous CdCl_2_ (99.99%), TeO_2_ powder (99%), Se powder (99%), sublimed sulfur (AR), 2,2′-Dithiobisbenzothiazole (98%), Tetraethylthiuram disulfide (97%), 1-Dodecanethiol (DDT, 98%), 3-Mercaptopropionic acid (99%), Zinc acetate dehydrate (99.99%), Ethanolamine (99%), 2-methoxyethanol (99.8%), Oleic acid (OA, AR), Oleylamine (OLA, 80%), and 1-Octadecene (ODE, 90%) were purchased from Alfa Aesar. All other chemicals and solvents were used as received.

### 2.2. Synthesis Method of CdTe NCs

In a typical synthesis procedure, 1 mmol TeO_2_ powder is dissolved in DDT (3 mL) and stirred for 3 min at room temperature, obtaining a yellow solution. OLA (3 mL) is injected into the above TeO_2_ mixture under N_2_ atmosphere; the solution turns black rapidly and the OLA-Te precursor is obtained. 2 mmol CdCl_2_ is dissolved in OLA and loaded into a three-neck bottle. The mixture is heated to 220 °C under N_2_ atmosphere and this temperature is maintained for 15 min until a clear solution is obtained. After that, the mixture is rapidly heated to 240 °C and the OLA-Te precursor is quickly injected into the mixture. To investigate the growth dynamics of CdTe NCs, aliquots of solution are taken from the reaction flask at regular time intervals and diluted with toluene for UV-vis absorption (UV) and photoluminescence (PL) measurements. The final product was dispersed and washed three times with methanol and toluene. 

### 2.3. Synthesis of CdSe NCs and CdS NCs

The fabrication of CdSe and CdS NCs are based on previous reported methods [37,38] with optimal process. For CdSe NCs fabrication, cadmium myristate was selected as a single cadmium salt. In a typical synthetic procedure for the CdSe NCs, 1 mmol of cadmium myristate and 10.0 mL ODE were loaded into a flask. Following this, the reaction system was heated to 170 °C and kept at this temperature for 10 min to dissolve the cadmium myristate. The reaction was then cooled down to room temperature and 2 mmol Se power was introduced into the reaction mixture. The mixture was then heated up to 230 °C under N_2_ flow. 0.35 mL OA was injected into the flask after 2 min. Finally, the reaction was kept at 230 °C for 30 min and cooled down to room temperature. The final product was dispersed and washed three times with acetone, methanol, and toluene. Fabrication of CdS NCs: In this case, cadmium myristate was selected as cadmium salt, while cadmium acetate was used in the literature [38]. Moreover, no myristic acid was used in this case. In a typical process, 2 mmol cadmium myristate, 0.125 mmol tetraethylthiuram disulfide, 0.375 mmol 2,2’-dithiobisbenzothiazole, 1 mmol sulfur, and 50 mL ODE were loaded into a three-necked flask. The mixture was heated to 140 °C under N_2_ flow and maintained at this temperature for 1 h to obtain a clear solution. The mixture was then heated to 240 °C at a rate of 15°/min and kept at this temperature for 30 min and then cooled down to room temperature. The purify method for CdS NCs is the same as that for CdSe NCs. There are no phosphine mixtures used in the fabrication of CdS NCs and CdSe NCs listed above. 

### 2.4. Device Fabrication and Characterization

The CdTe NCs are dispersed in toluene with a concentration of 45 mg/mL. The CdSe NCs are dispersed into a chlorobenzene/pyridine (with volume ratio of 9:1) solvent, with a concentration of 43 mg/mL, while the CdS NCs are dispersed into a pyridine/n-propanol (with volume ratio of 1:1) solvent with a concentration of 36 mg/mL. In a typical process, solar cells with configuration of ITO/ZnO/CdSe/CdTe/Au or ITO/ZnO/CdS/CdTe/Au are fabricated by a layer-by-layer solution process. The Zn^2+^ precursor was prepared by dissolved 3.2925 g zinc acetate dehydrate into 30 mL 2-methoxyethanol with 0.905 mL ethanolamine and refluxed at 80 °C for 1 h under N_2_ flow. The mixture was collected into a vial after being cooled down to room temperature. Firstly, several drops of the Zn^2+^ precursor solution are deposited on ITO substrates and spin-casted at 3000 rpm for 30 s. Then the substrate is transferred to a hotplate and annealed at 400 °C for 10 min to eliminate any organic solvent and impurity. Then, several drops of CdSe or CdS NCs solution are deposited on the ITO/ZnO substrate and spin-casted at 3000 rpm for 20 s. After that, the substrate is transferred to a hot plate and annealed at 150 °C for 10 min and 350 °C (CdSe NCs) or 380 °C (CdS NCs) for 40 s. Several drops of the CdTe NCs solution (dissolved into toluene at a concentration of 48 mg/mL) are deposited on the ITO/ZnO/CdSe (or CdS) substrate and spin-casted at 1100 rpm for 20 s. The substrate is then transferred to a hotplate and annealed at 150 °C for 3 min. Following this, the substrate is dipped into saturated CdCl_2_/methanol solution for 10 s and rinsed with n-propyl and quickly putted on the hotplate at 360 °C for 40 s. Finally, five layers of CdTe NCs are deposited on the ITO/ZnO/CdS (or CdSe) via a similar layer-by-layer sintering process. The detail fabricating process can be found in our previously reported works [13]. Finally, Au (~80 nm) back contact electrodes are deposited via thermal vacuum evaporation through a shadow mask with an active area of 0.16 cm^2^. The morphology of NC is characterized by transmission electron microscopy (TEM, FEI Tecnai G2 F20, FEI, OR, USA) and atomic force microscopy (AFM, DI/MutiMode, Veeco Instruments, Inc., Plainview, MN, USA), while the structure is characterized by X-ray diffraction (XRD, X’pert Pro M, Philips, Amsterdam, The Netherlands). A step profiler (Dektak XT10, Bruker, Karlsruhe, Germany) is used to measure the thickness of the NC thin film. The optical properties and electronic properties of the NC devices are investigated by using a solar simulator (XES-40S1, San-Ei Electric Co., Ltd., Osaka, Japan), UV-Vis (UV-5100B, Shanghai Metash Instrument Co., Ltd., Shanghai, China) and photo-luminescence (PL, FL3-2IHR, HORIBA Instruments Inc., Irvine, CA, USA), and external quantum efficiency (EQE, Solar Cell Scan100, Zolix Instruments Co., Ltd., Beijing, China).

## 3. Result and Discussion

**Figure 1:**
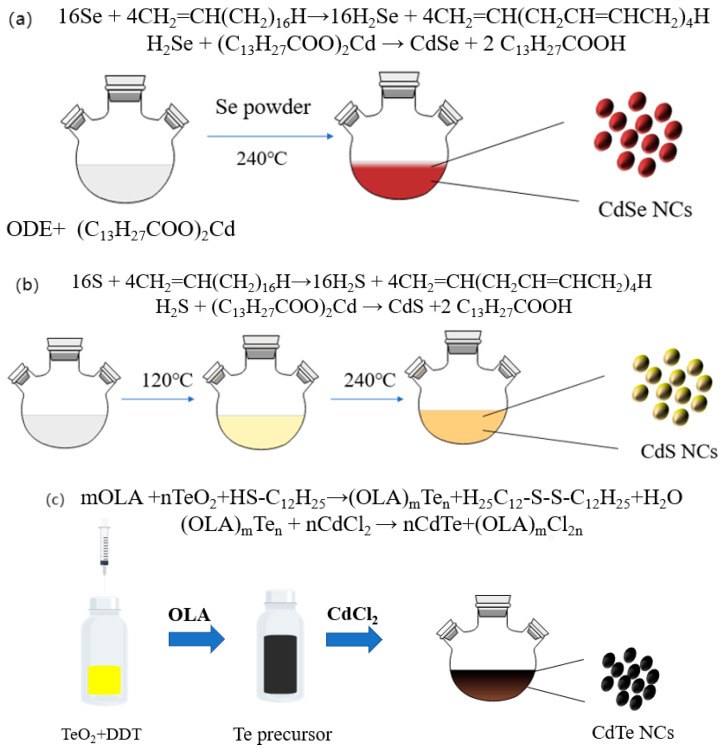
A schematic diagram of the NC fabricating process (**a**) CdSe, (**b**) CdS, (**c**) CdTe NCs.

In this research, CdS, CdSe, and CdTe NCs are synthesized using phosphine-free receipt. In the case of CdSe and CdS NCs, ODE is selected as a non-coordinating solvent and the synthesis processes are non-injection methods. As shown in Figure 1a, Se powder is used as a Se precursor, while cadmium carboxyl (cadmium myristate) is used as a Cd^2+^ precursor. At a temperature of 230 °C, H^+^ would be supplied in situ by ODE and react with Se to form H_2_Se, as reported before [37]. CdSe NCs will be formed because of the reaction between H_2_Se and cadmium myristate. As myristic acid is generated and acts as ligands capping on the surface of CdSe NCs during the reaction, NCs with homogeneous size are obtained. In the case of synthesizing CdS NCs, as the activity of sulfur is low when comparing to the Cd ion, the addition of the 2,2′-Dithiobisbenzothiazole initiator ensures the activity of S precursor and guarantees the formation of high-quality CdS NCs (Figure 1b). For CdTe NCs (Figure 1c), however, a similar non-injection method will not work. Because of the low activity of Te element, Te powder cannot be used directly as a Te precursor for CdTe NCs’ fabrication. An efficient phosphine-free Te precursor is essential to fabricate high-quality CdTe NCs. To overcome this difficulty, a Te precursor is prepared by reducing TeO_2_ powder with DDT in the presence of OLA to generate a soluble alkylammonium telluride at room temperature. CdTe NCs can then be prepared by injecting this phosphine-free Te precursor into the CdCl_2_/OLA mixture at a temperature of 240 °C and refluxed at 220 °C for 30 min. It should be noticed that the NCs fabricated by this phosphine-free receipt is both economical and environmentally-friendly. The chemicals’ prices for fabricating 1 g CdS, CdSe, and CdTe NCs under different methods are presented in Tables S1 and S2. Using our phosphine-free receipt, the cost for fabricating 1g CdS, CdSe, and CdTe NCs is $58.96, $16.90, and $18.33, respectively, while the cost of NCs synthesized by conventional methods are $94.11, $71.63, and $61.29 respectively. It is obvious that our phosphine-free receipt can significantly decrease the cost of NC photovoltaic products and is suitable for industrial mass production.

**Figure 2:**
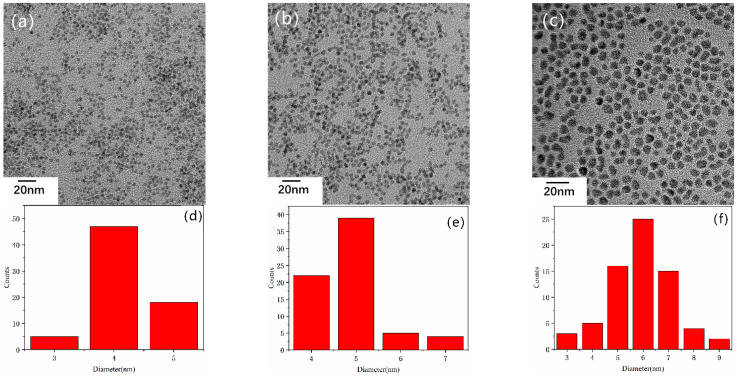
TEM images of (**a**) CdS (**b**) CdSe (**c**) CdTe NCs. (Scale bar 20 nm), particle size distribution of (**d**) CdS (**e**) CdSe (**f**) CdTe NCs.

To understand the properties of the NCs synthesized by our phosphine-free method, various characterizations were carried out. Figure 2 shows the transmission electron microscope (TEM) images of the as-prepared CdS (Figure 2a), CdSe (Figure 2b), and CdTe (Figure 2c) NCs. All the NCs exhibit a spherical shape. To investigate the uniformity of the size of NCs, a statistical number of 100 is selected for each NCs. As shown in Figure 2d–f, the size of all the NCs is homogeneous with mean size of 4.2 nm, 4.9 nm, and 5.9 nm for CdS, CdSe, and CdTe NCs, respectively. This result shows that our phosphine-free synthesis method allows effective control of the size and shape of the nanocrystals. We speculate that the origin of this uniformity is the in situ formation of carboxyl acid during the reaction, which has been confirmed in our previous work [39].

**Figure 3:**
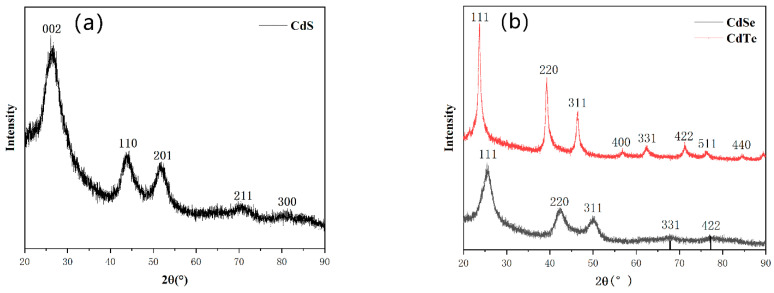
The XRD pattern of the (**a**) CdS (**b**) CdSe, and CdTe NCs.

The crystal structures of the as-prepared CdS, CdSe, and CdTe NCs products are characterized by X-ray diffraction (XRD) (Figure 3). The XRD pattern of spherical CdS NCs exhibited diffraction peaks at about 25.1°, 41.8°, and 49.6° (Figure 3a), corresponding to the (111), (311), and (331) planes of the zinc-blende crystal structure of CdS. For CdSe NCs, diffraction patterns with peaks at about 25.3°, 42.0°, 49.7°, 67.1°, and 76.3° could be identified, corresponding to the (111), (220), (311), (331), and (422) facets of zinc–blende CdSe NCs [39]. A similar zinc–blende structure is revealed by the XRD pattern of CdTe NCs (Figure 3b). It is noted that the half peak width of CdTe (the main peak (111)) is narrower than that of CdSe or CdS NCs. As the CdS, CdSe, and CdTe NCs are collected by washing the NCs products with acetone, methanol, and toluene, no further treatment such as reflowing is carried out; the residual metal precursor or other chemical may affect the width of XRD peaks. Therefore, we speculate that the uniformity of the nanoparticles, or their size may not be related to the width of the XRD peaks.

**Figure 4:**
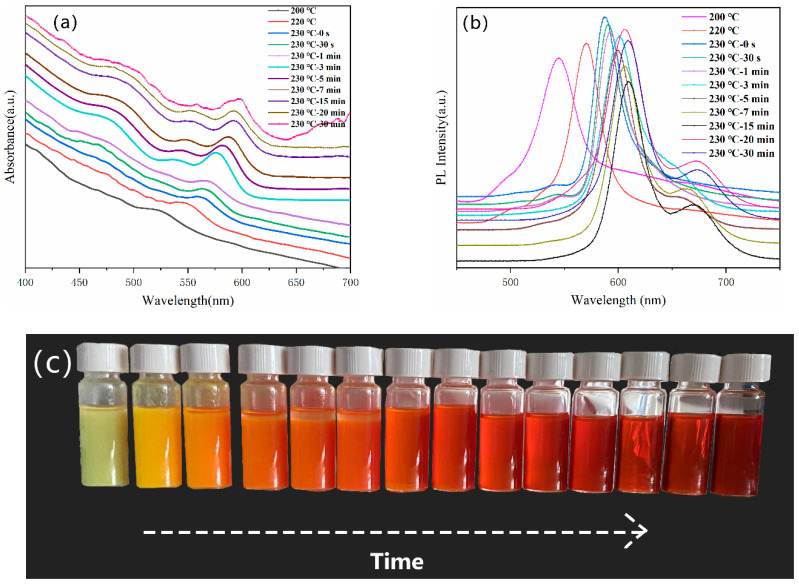
(**a**) UV absorbance of CdSe NCs with different growth time; (**b**) PL spectra of the CdSe NCs with different growth time; (**c**) PL images with excitation by 365 nm UV light.

The growing process is of great importance to control the optoelectronic properties of NCs as particle size has a significant influence on their electronic band structures. To monitor the growth kinetics of NCs, we characterized the UV–vis absorption and PL (Photoluminescence) spectra for different growth times after the formation of semiconductor NCs. For instance, Figure 4 showed the temporal evolutions of the UV–vis (Figure 4a) and PL spectra (Figure 4b) of CdSe NCs prepared by the phosphine-free non-injection method. As shown in Figure 2a, after the reaction temperature reached 220 °C, CdSe NCs are formed and the absorption peak is located at 543 nm, while the PL peak is at 572 nm. The reaction temperature is maintained at 230 °C and several drops of the NCs solution are taken out at different time intervals. After crystal nucleus formation, the absorption and PL peaks shift to 564 nm and 594 nm, respectively, after growing for 0.5 min. Although the absorption/PL peaks undergo a redshift to longer wavelengths as growing time increases, this trend slows down when the reaction progressed from 7 min to 30 min, which could be seen in the UV–vis (Figure 4a) and PL spectra (Figure 4b). In addition, from Figure 4b, one can see that the PL peaks are sharp and narrow with half-band width around 37 nm after the reaction time reaches 7 min, which implies a narrow NCs size distribution. Therefore, the phosphine-free method is promising for high-quality CdSe NCs fabrication; similar results can be found in the case of CdTe NCs (Figure S1). However, although a redshift is also found in the case of CdS NC (Figure S2) PL main peaks, the PL shows very broad emissions at high wavelengths and more mixed peaks appear in this case. We speculate that there are more defects in the CdS NCs and/or the effects of other chemicals such as 2,2′-Dithiobisbenzothiazole, tetraethylthiuram disulfide. The changes in fluorescent properties of NC with different reaction times are also monitored by using a portable UV light source. As shown in Figure 4c, the PL is yellow and green color at the beginning of reaction. Then the color slowly turns to yellow, light red, and to deep red, which implies that the NCs grow into larger sizes. After 20 min, there are almost no changes in the NC color, suggesting their low monomer concentration. The final size of CdS, CdSe, and CdTe NCs are calculated to be 1.92 nm, 4.50 nm, and 10.51 nm by using an experience formula [17]:

D = (1.6122 × 10 ^−9^) λ^4^ − (2.6575 × 10 ^−6^) λ^3^ + (1.6242 × 10 ^−3^) λ^2^ − (0.4277) λ + (41.57)

Therefore, the calculated value for CdSe NC conforms to the value from the TEM measurement. However, the calculated value for CdS or CdTe NCs is quite different from the TEM results. We speculate that this formula is suitable for predicting the size of CdSe NCs but not for CdS or CdTe NCs. Moreover, a cadmium precursor or other chemical may affect the absorption of CdS or CdTe NCs; more work needs to be carried out to clarify this. 

**Figure 5:**
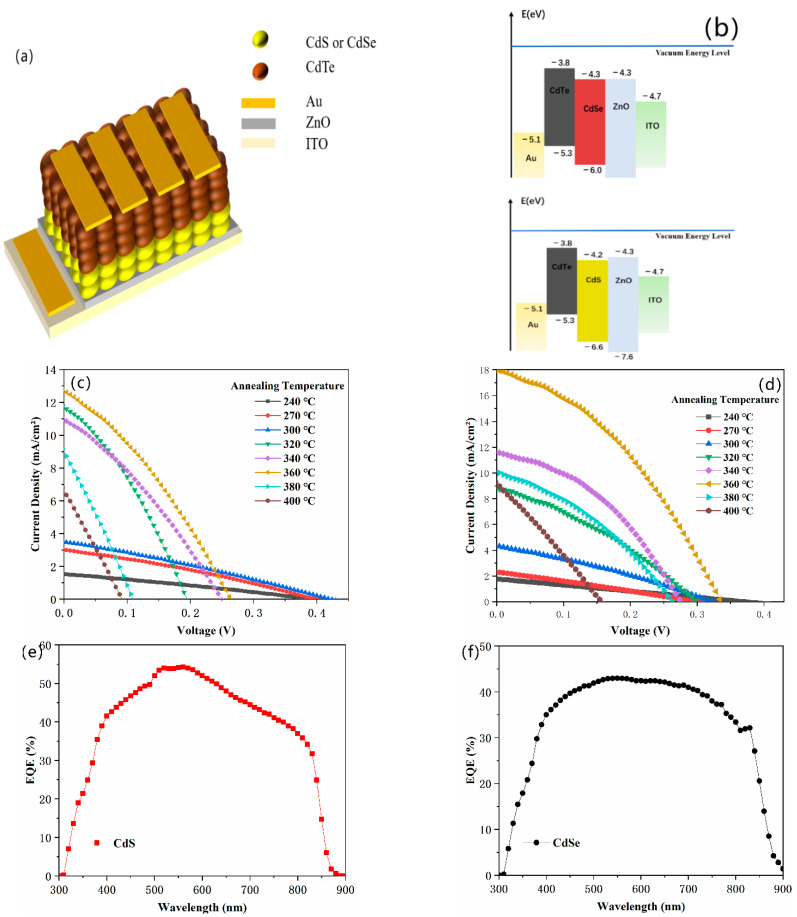

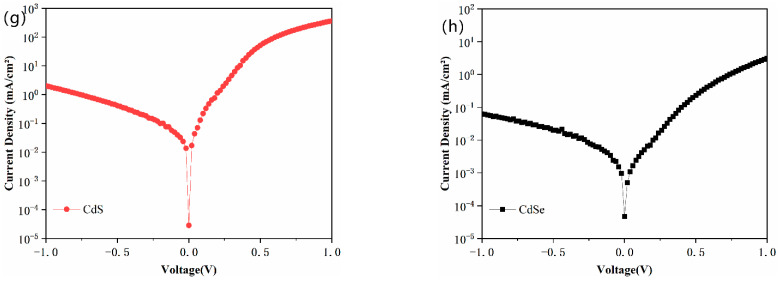
(**a**) The NC solar cell configuration; (**b**) Band alignment of the NC solar cell; *J-V* curves of (**c**) ITO/ZnO/CdSe/CdTe/Au and (**d**) ITO/ZnO/CdS/CdTe/Au with different annealing temperatures under light with different annealing temperatures under light. The corresponding EQE spectra of (**e**) ITO/ZnO/CdS/CdTe/Au (**f**) ITO/ZnO/CdSe/CdTe/Au; *J*-*V* curves of (**g**) ITO/ZnO/CdS/CdTe/Au and (**h**) ITO/ZnO/CdSe/CdTe/Au under dark.

**Figure 6:**
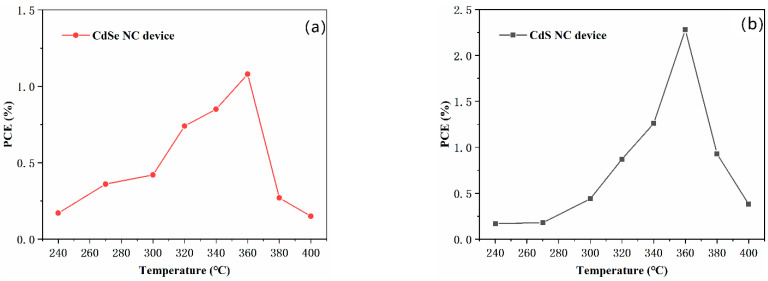
Evolution of parameters PCE for (**a**) CdTe/CdSe NC devices (**b**) CdTe/CdS NC devices under different annealing temperatures.

To exhibit the potential of our phosphine-free semiconductor NCs in the field of optoelectronic devices, photovoltaic devices were fabricated based on these materials. Devices with an inverted structure of ITO/ZnO (40 nm)/CdSe (60 nm)/CdTe (~400 nm)/Au (80 nm), and ITO/ZnO (40 nm)/CdS (60 nm)/CdTe (~400 nm)/Au (80 nm) were fabricated through a layer-by-layer spin-coating process, as reported before [38,39]. The device structure and band alignment with CdS NCs and CdSe NCs as *n*-type materials and CdTe as a *p*-type material are presented in Figure 5a,b. In this experiment, the CdTe NCs solution is dissolved into toluene at a concentration of 40 mg/mL, which is different from the works reported before [40]. For CdTe NCs solar cells, an appropriate annealing temperature is essential to increase the grain size and eliminate interface defects in the CdTe NCs thin film. From the AFM image presented in Figure S3, the grain size of CdTe NCs is up to several hundred nanometers, while the thickness of CdTe NCs is ~389.9 nm (characterized by using a step profiler). The *J*-*V* curves for the CdTe NC solar cells with CdSe and CdS NCs as the *n*-type partner under different annealing temperatures are exhibited in Figure 5c,d, while the photovoltaic parameters are as shown in Figure S4. The PCE of CdSe and CdS NC devices with different annealing temperatures is also summarized in Figure 6a,b. In the case of CdSe/CdTe junction solar cells, short circuit current (*J_sc_*) increases almost linearly from 1.54 mA/cm^2^ to 12.74 mA/cm^2^ when the annealing temperature rises from 240 to 360 °C. When the annealing temperature further increases, the *J_sc_* drops linearly. Similar laws are found for the changes in PCE of NCs solar cells. The *V*_oc_ of devices are around 0.4 V at low annealing temperatures (below 300 °C ), while *V*_oc_ below 0.3 V is obtained for annealing temperatures higher than 300 °C. The champion device is obtained at an annealing temperature of 360 °C; it exhibited a short circuit current density (*J*_sc_) of 12.74 mA/cm^2^, an open circuit voltage (*V*_oc_) of 0.26 V, a fill factor (FF) of 32.35%, and a PCE of 1.08%. This value is significantly lower than those ever reported (5.81%) with the same device structure (NCs are fabricated by using phosphine mixture) and similar annealing strategy [26,40]. The low device performance is mainly attributed to the low active layer (CdTe/CdSe) quality. We speculate that, in the case of CdTe NC solar cells with CdSe NC as *n*-type partner, the CdSe NCs is capped with oleic acid. During the heat-treatment of the CdSe NC film, the oleic acid cannot be removed effectively. The oleic acid is insulating and acts as a carrier recombination center, which will drastically decrease the device performance. When CdS NCs are used as *n*-type partners for CdTe NC solar cells, the trend of how devices performance changes with annealing temperature are similar to that of CdSe NCs devices. The *V*_oc_ of devices are kept stable at around 0.3–0.4 V when annealing temperature is below 360 °C. The PCE increases linearly when annealing temperature rises from 240 to 360 °C. At low annealing temperature (below 320 °C ), the PCE is less than 1%. On the contrary, devices annealed at a moderate temperature of 340–360 °C show optimal performance. At a temperature of 360 °C, we obtain our champion device, which shows the following merits: a short circuit voltage (*J*_sc_) of 18.01 mA/cm^2^, an open circuit voltage (*V*_oc_) of 0.33 V, a fill factor (FF) of 37.84% and a PCE of 2.28%. This value is two times higher than that for CdSe NC devices. It is noted that the defects of CdS NCs are higher than that of CdSe NCs (before purify). However, as no long chain alky acid ligands are used for the fabrication of CdS NCs, during the sintering process (the NCs thin film fabrication), the CdS NCs’ thin films are more compact and low interface defects between CdS NCs is obtained. On the other hand, as OA cannot be removed completely during the sintering process (OA is insulated material), more defects are existed between CdSe NCs. Therefore, low carrier recombination is attained in the case of CdS NC solar cells, which will lead to higher *J*_sc_ and PCE. Both NC devices (with CdSe of CdS NCs as *n*-type partner) decay at higher annealing temperatures. This may be due to oxidation of the CdTe NC film at high temperatures, which has been confirmed before [38]. When comparing devices based on NCs fabricated by the traditional method [38,39] with phosphine mixture, the series resistance (*R*_s_) obtained here (Table 1 and Table 2) is several times higher than those reported before. The high *R*_s_ will result in a higher carrier recombination in the active layer and decrease the FF of the NC devices. Further investigation should be carried out to further increase the quality of the NC active layer. Figure 5e,f show the EQE spectra of the CdS NC and CdSe NC champion devices. Comparing to the CdSe NC device, the CdS NC device has a better EQE response at wavelengths from 400 nm to 800 nm, implying that the CdS NC device has a better capability to transfer photons to valence electrons and generate electron–hole pairs than that of the CdSe NC device. When the EQE curves are integrated, the calculated *J_sc_* of 16.57 mA/cm^2^ (for CdS NC device) and 14.45 mA/cm^2^ (for CdSe NC device) are predicted, which are consistent with the *J_s_*_c_ value from the *J-V* curves under light (Figure 5e,f). From the dark *J*–*V* curves for CdSe NC and CdS NC devices (Figure 5g,h), it is evident that typical diode properties are obtained in both cases. 

In summary, CdS, CdSe, and CdTe NCs are fabricated successfully by a phosphine-free receipt. NCs with homogeneous morphology and size can be well controlled by this method. Based on CdS, CdSe, and CdTe NCs, CdTe NC solar cells with configuration of ITO/ZnO/CdSe/CdTe/Au and ITO/ZnO/CdS/CdTe/Au were fabricated and investigated. It was found that at optimal annealing temperature, we obtain champion devices with PCE of 1.08% and 2.28% by using CdSe and CdS NC as *n*-type partners, respectively. We believe that by optimizing device-fabricating techniques (such as using ligands exchange technology, designing active layer thickness, etc.), the PCE of these NC devices can be further improved. With a low-cost and environmentally-friendly fabricating process, these NC solar cells may pave the way for next-generation photovoltaic devices. 

## Figures and Tables

**Table 1:** Summarized performance of CdTe/CdSe NC solar cells with different annealing temperatures (Figure 5a).

Annealing Temperature (℃)	PCE (%)	*J*_sc_ (mA/cm^2^)	FF (%)	*V*_oc_ (V)	*R*_s_ (Ω·cm^2^)	*R*_sh_ (Ω·cm^2^)
240	0.17 (± 0.02)	1.54 (± 0.2)	27.27 (± 2)	0.40 (± 0.02)	1330.42	1671.38
270	0.36 (± 0.05)	3.03 (± 0.5)	29.68 (± 4)	0.40 (± 0.03)	611.66	1458.51
300	0.42 (± 0.04)	3.52 (± 0.2)	28.35 (± 3)	0.42 (± 0.02)	649.64	904.81
320	0.74 (± 0.02)	12.04 (± 0.4)	30.32 (± 3)	0.20 (± 0.01)	74.16	176.07
350	0.85 (± 0.02)	11.01 (± 0.5)	30.93 (± 2)	0.25 (± 0.03)	98.84	234.84
360	1.08 (± 0.05)	12.74 (± 0.3)	32.35 (± 4)	0.26 (± 0.02)	93.84	334.76
380	0.27 (± 0.012)	9.16 (± 0.4)	25.93 (± 1)	0.11 (± 0.02)	60.89	84.61
400	0.15 (± 0.02)	6.80 (± 0.2)	24.39 (± 2)	0.09 (± 0.01)	87.54	86.20

**Table 2:** Summarized performance of CdTe/CdS NC solar cells with different annealing temperatures (Figure 5b).

Annealing Temperature (°C)	PCE (%)	*J*_sc_ (mA/cm^2^)	FF (%)	*V*_oc_ (V)	*R*_s_ (Ω cm^2^)	*R*_sh_ (Ω·cm^2^)
240	0.17 (± 0.02)	1.77 (± 0.3)	25.26 (± 2)	0.37 (± 0.02)	1306.76	1500.15
270	0.18 (± 0.01)	2.32 (± 0.2)	26.63 (± 3)	0.30 (± 0.03)	721.03	1103.55
300	0.44 (± 0.02)	4.40 (± 0.5)	30.36 (± 3)	0.33 (± 0.02)	337.50	781.55
320	0.87 (± 0.03)	8.81 (± 0.3)	33.12 (± 3)	0.30 (± 0.03)	145.38	670.95
340	1.26 (± 0.02)	11.64 (± 0.6)	39.61 (± 3)	0.27 (± 0.01)	77.86	611.93
360	2.28 (± 0.02)	18.01 (± 0.6)	37.84 (± 4)	0.33 (± 0.02)	70.40	600.15
380	0.93 (± 0.01)	10.13 (± 0.3)	34.91 (± 3)	0.26 (± 0.02)	97.51	315.32
400	0.38 (± 0.2)	9.29 (± 0.2)	26.84 (± 2)	0.15 (± 0.03)	105.35	113.92

## Data Availability

Not applicable.

## Supplementary Materials

The following are available online at https://www.mdpi.com/article/10.3390/nano11082071/s1, Table S1. The price of chemicals from the website. Table S2. The cost of materials for fabricating 1g CdS, CdSe, and CdTe NCs with different methods. Figure S1. (a) UV absorbance of CdTe NCs with different growth times; (b) PL emission spectra of the CdTe NCs with different growth times; (c) PL images with excitation by 365 nm UV light. Figure S2. (a) UV absorbance of CdS NCs with different growth times; (b) PL emission spectra of the CdS NCs with different growth times. Figure S3. Atomic for microscopy (AFM) images of devices. Figure S4. The microscopy image of a NC solar cell (one single substrate containing 5 devices with active areas of 0.16 cm²).
