# Supplementary material for: Synthesis of Group II-VI Semiconductor Nanocrystals via Phosphine Free Method and Their Application in Solution Processed Photovoltaic Devices
Source: Nanomaterials (Basel). 2021 Aug 15;11(8):2071. doi: 10.3390/nano11082071 (PMC8399757; doi:10.3390/nano11082071)
Supplement: Supplementary file 1 [file nanomaterials-11-02071-s001.zip › nanomaterials-1335041-supplementary.pdf]

nanomaterials

Supplementary Materials

Synthesis of Group II-VI Semiconductor Nanocrystals via Phosphine Free Method and their Application in Solution Processed Photovoltaic Devices

Mingyue Hou [1], Zhaohua Zhou [1], Ao Xu [1], Kening Xiao [1], Jiakun Li [1], Donghuan Qin [1,2,*], Wei Xu [1,2,*] and Lintao Hou [3,*]

[1] School of Materials Science and Engineering, South China University of Technology, Guangzhou 510640, China; 201830140083@mail.scut.edu.cn (M.H.); 201830320430@mail.scut.edu.cn (Z.Z.); 202020118759@mail.scut.edu.cn (A.X.); 201830320324@mail.scut.edu.cn (K.X.); 201830640170@mail.scut.edu.cn (J.L.)

[2] State Key Laboratory of Luminescent Materials & Devices, Institute of Polymer Optoelectronic Materials & Devices, South China University of Technology, Guangzhou 510640, China

[3] Guangdong Provincial Key Laboratory of Optical Fiber Sensing and Communications, Guangzhou Key Laboratory of Vacuum Coating Technologies and New Energy Materials, Siyuan Laboratory, Department of Physics, Jinan University, Guangzhou 510632, China

* Correspondence: qindh@scut.edu.cn (D.Q.); xuwei@scut.edu.cn (W.X.); thlt@jnu.edu.cn (L.H.)

Table S1. The price of chemicals from the website: www.alfa.com.(13 July 2021)

Chemicals	Price
Anhydrous $CdCl_2$ (99.99%)	$ 481.66/100 g
TeO_2 powder (99%)	$ 542.76/100 g
Tellurium powder (99.999%)	$ 444.08/100 g
Se powder (99%)	$ 48.41/50 g
Sulfur powder (99.5%)	$ 95.74/500 g
2,2′-Dithiobis (benzothiazole)(98%)	$ 14.08/100 g
Myristic acid (99%)	$ 51.97/500 g
Sodium hydroxide (pellets, 98%)	$ 42.85/500 g
Cadmium nitrate tetrahydrate (99.9%)	$ 123.43/50 g
1-Dodecanethiol (DDT, 98%)	$ 30.16/100 g
Tetraethylthiuram disulfide (97%)	$ 43/100 g
Oleylamine (OLA, 80%)	$ 8.35/100 mL
1-Octadecene (ODE, 90%)	$ 26.91/250 mL
Oleic acid (OA, AR)	$ 68.68/1000 mL
Trioctylphosphine (TOP,90%)	$ 245.78/100 mL
Trioctylphosphine oxide (TOPO, 98%)	$ 252.74/100 g

Table S2. The materials cost for fabricating 1 g CdS, CdSe and CdTe NCs with different method.

NCs (1 g)	Traditional Method with Phosphine	Phosphine-free Method
CdTe	$ 61.29	$ 18.33
CdSe	$ 71.63	$ 16.90
CdS	$ 94.11	$ 58.96

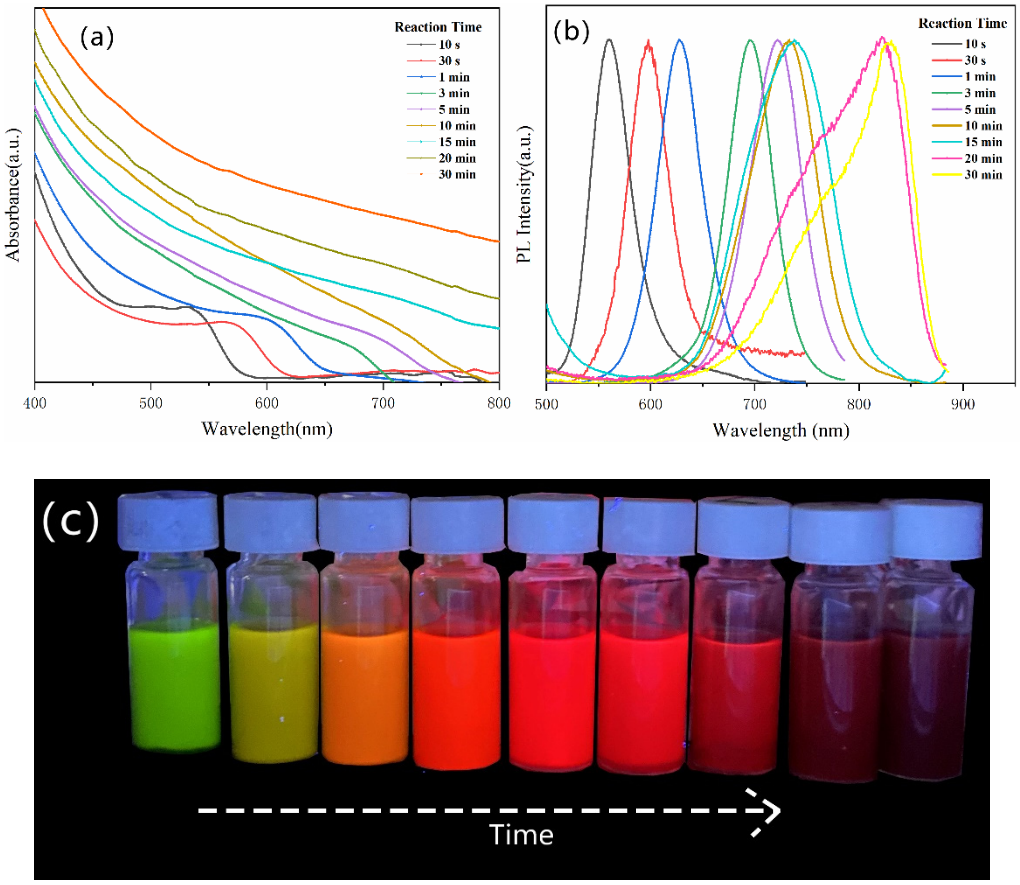

Figure S1. (a) UV absorbance of CdTe NCs with different growth time; **(b)** PL emission spectra of the CdTe NCs with different growth time; **(c)** PL images with excitation by 365 nm UV light.

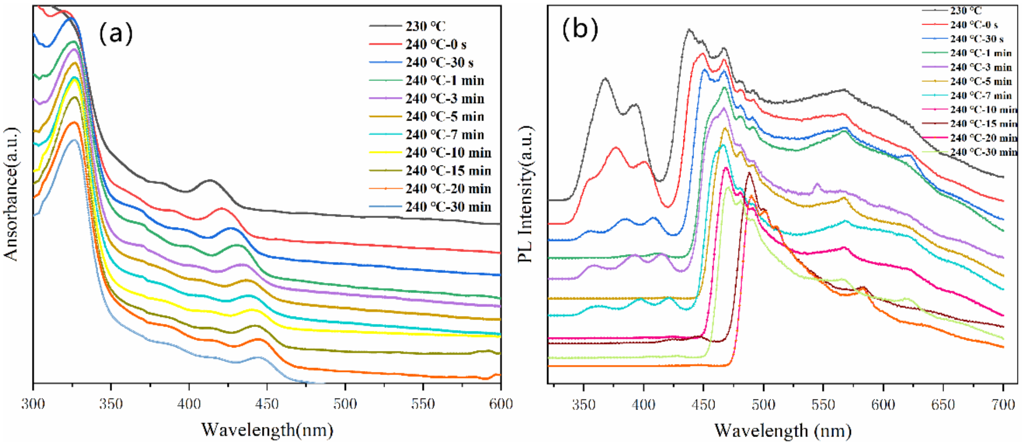

Figure S2. (a) UV absorbance of CdS NCs with different growth time; **(b)** PL emission spectra of the CdS NCs with different growth time.

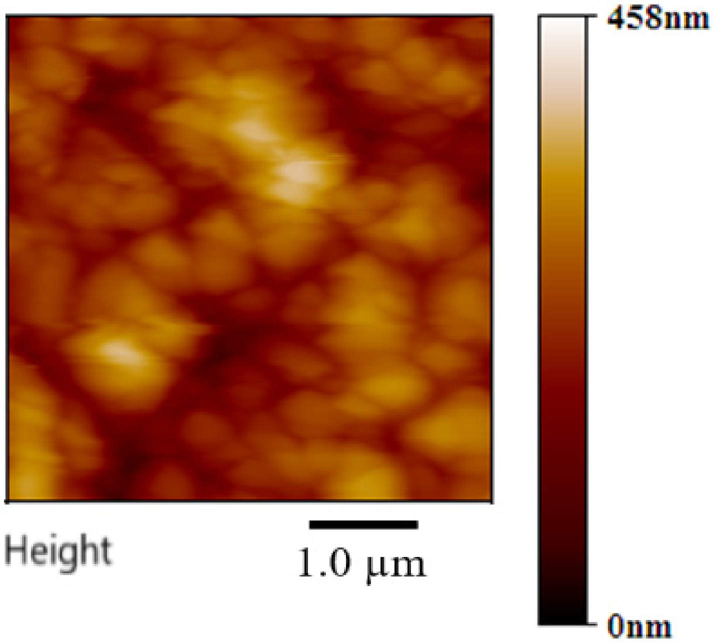

Figure S3. Atomic for microscopy (AFM) images of devices.

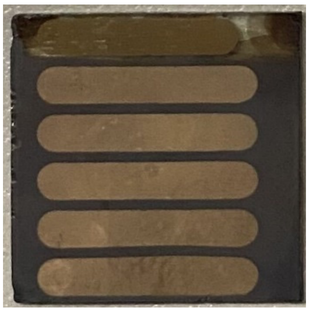

Figure S4. The microscopy image of a NC solar cell (one single substrate containing 5 devices with active areas of 0.16cm²).
